# Informative Gene Selection and Direct Classification of Tumor Based on Chi-Square Test of Pairwise Gene Interactions

**DOI:** 10.1155/2014/589290

**Published:** 2014-07-23

**Authors:** Hongyan Zhang, Lanzhi Li, Chao Luo, Congwei Sun, Yuan Chen, Zhijun Dai, Zheming Yuan

**Affiliations:** ^1^Hunan Provincial Key Laboratory of Crop Germplasm Innovation and Utilization, Changsha 410128, China; ^2^College of Information Science and Technology, Hunan Agricultural University, Changsha 410128, China; ^3^Hunan Provincial Key Laboratory for Biology and Control of Plant Diseases and Insect Pests, Changsha 410128, China

## Abstract

In efforts to discover disease mechanisms and improve clinical diagnosis of tumors, it is useful to mine profiles for informative genes with definite biological meanings and to build robust classifiers with high precision. In this study, we developed a new method for tumor-gene selection, the Chi-square test-based integrated rank gene and direct classifier (*χ* ^2^-IRG-DC). First, we obtained the weighted integrated rank of gene importance from chi-square tests of single and pairwise gene interactions. Then, we sequentially introduced the ranked genes and removed redundant genes by using leave-one-out cross-validation of the chi-square test-based Direct Classifier (*χ* ^2^-DC) within the training set to obtain informative genes. Finally, we determined the accuracy of independent test data by utilizing the genes obtained above with *χ* ^2^-DC. Furthermore, we analyzed the robustness of *χ* ^2^-IRG-DC by comparing the generalization performance of different models, the efficiency of different feature-selection methods, and the accuracy of different classifiers. An independent test of ten multiclass tumor gene-expression datasets showed that *χ* ^2^-IRG-DC could efficiently control overfitting and had higher generalization performance. The informative genes selected by *χ* ^2^-IRG-DC could dramatically improve the independent test precision of other classifiers; meanwhile, the informative genes selected by other feature selection methods also had good performance in *χ* ^2^-DC.

## 1. Introduction

Tumors are the consequences of interactions between multiple genes and the environment. The emergence and rapid development of large-scale gene-expression technology provide an entirely new platform for tumor investigation. Tumor gene-expression data has the following features: high dimensionality, small or relatively small sample size, large differences in sample backgrounds, presence of nonrandom noise (e.g., batch effects), high redundancy, and nonlinearity. Mining of tumor-informative genes with definite biological meanings and building of robust classifiers with high precision are important goals in the context of clinical diagnosis of tumors and discovery of disease mechanisms.

Informative gene selection is a key issue in tumor recognition. Theoretically, there are 2^*m*^ possibilities in selecting the optimal informative gene subset from *m* genes, which is an N-P hard problem. Available high-dimensional feature-selection methods often fall into one of the following three categories: (i) filter methods, which simply rank all genes according to the inherent features of the microarray data, and their algorithm complexities are low. However, redundant phenomena are usually present among the selected informative genes, which may result in low classification precision. Univariate filter methods include *t*-test [1], correlation coefficient [2], Chi-square statistics [3], information gain [4], relief [5], signal-to-noise ratio [6], Wilcoxon rank sum [7], and entropy [8]. Multivariable filter methods include mRMR [9], correlation-based feature selection [10], and Markov blanket filter [11]; (ii) wrapper methods, which search for an optimal feature set that maximizes the classification performance, defined in terms of an evaluation function (such as cross-validation accuracy). Their training precision and algorithm complexity are high; consequently, it is easy for over-fitting to occur. Search strategies include sequential forward selection [12], sequential backward selection [12], sequential floating selection [13], particle swarm optimization algorithm [14], genetic algorithm [15], ant colony algorithm [16], and breadth-first search [17]. SVM and ANN are usually used for feature subset evaluation; (iii) embedded methods, which use internal information about the classification model to perform feature selection. These methods include SVM-RFE [18], support vector machine with RBF kernel based on recursive feature elimination (SVM-RBF-RFE) [19], support vector machine and T statistics recursive feature elimination (SVM-T-RFE) [20], and random forest [21].

Classifier is another key issue in tumor recognition. Traditional classification algorithms include Fisher linear discriminator, Naive bayes (NB) [22], K-nearest neighbor (KNN) [23], DT [24], support vector machine (SVM) [18], and artificial neural network (ANN) [25]. There are dominant expressions in parametric models (e.g., Fisher linear discriminator) based on induction inference. The first goal for parametric models is to obtain general rules through training-sample learning, after which these rules are utilized to judge the testing sample. However, this is not the case for nonparametric models (e.g., SVM) based on transduction inference, which predict special testing samples through observation of special training samples, but classifiers needed for training. Training is the major reason for model over-fitting [3]. Therefore, it is important to determine whether it is feasible to develop a direct classifier based on transduction interference that has no demand for training.

In recent years, several methods have been developed to perform both feature-selection and classification for the analysis of microarray data as follows: prediction analysis for microarrays (PAM), based on nearest shrunken centroids [26]; top scoring pair (TSP), based entirely on relative gene expression values [27]; refined TSP algorithms, such as k disjoint Top Scoring Pairs (*k*-TSP) for binary classification and the HC-TSP, HC-*k*-TSP for multiclass classification [28]; an extended version of TSP, the top-scoring triplet (TST) [29]; an extended version of TST, top-scoring “N” (TSN) [30]. A remarkable advantage of the TSP family is that they can effectively control experimental system deviations, such as background differences and batch effects between samples. However, TSP,* k*-TSP, TST, and TSN are only suitable for binary data, and the HC-TSP/HC-TSP calculation process for conversion from multiclass to binary classification is tedious. The gene score Δ_*ij*_ [27] cannot reflect size differences among samples, and k-TSPs may introduce redundancy and undiscriminating voting weights.

Chi-square-statistic-based top scoring genes (TSG) [31], an improved version of TSP family we proposed before, introduces Chi-square value as the score for each marker set so that the sample size information is fully utilized. TSG proposes a new gene selection method based on joint effects of multiple genes, and the informative genes number is allowed both even and odd. Moreover, TSG gives a new classification method with no demand for training, and it is in a simple unified form for both binary and multiclass cases. In TSG paper, we did not name the classification method alone. Here we called it the chi-square test-based direct classifier (*χ* ^2^-DC). To predict the class information for each sample in the test data, *χ* ^2^-DC use the selected marker set and calculate the scores of this sample belonging to each class. The predicted class is set to be the one that has the largest score. Although TSG has many merits, it also has the following disadvantages: (i) for *k* ≥ 3, in order to find the top scoring *k* genes (TS_*k*_), all the combined scores between TS_*k*-1_ and each of remaining gene need to be calculated. It needs a large amount of calculation; (ii) if there are multiple TS_*k*_s with identical maximum Chi-square value, TSG should further calculate the LOOCV accuracy of these TS_*k*_s using the training data and record those TS_*k*_s that yield the highest LOOCV accuracy. If there is still more than one TS_*k*_, the computational complexity will be much higher to find TS_*k*+1_; (iii) in TSG, an upper bound *B* should be set and find TS_*B*_. However, the number of information genes is often less than *B*. The termination condition of feature selection is not objective enough.

Emphasizing interactions between genes or biological marks is a developing trend in cancer classification and informative gene selection. The TSP family, mRMR, doublets [32], nonlinear integrated selection [33], binary matrix shuffling filter (BMSF) [34], and TSG all take interactions into consideration. In genome-wide association studies, ignorance of interactions between SNPs or genes will cause the loss of inheritability [35]. Therefore, we developed a novel high-dimensional feature-selection algorithm called a Chi-square test-based integrated rank gene and direct classifier (*χ* ^2^-IRG-DC), which inherits the advantages of TSG while overcoming the disadvantages documented above in feature selection. First, this algorithm obtains the weighted integrated rank of gene importance on the basis of chi-square tests of single and pairwise gene interactions. Then, the algorithm sequentially forward introduces ranked genes and removes redundant parts using leave-one-out cross validation (LOOCV) of *χ* ^2^-DC within the training set to obtain the final informative gene subset of tumor.

A large number of feature-selection methods and classifiers currently exist. Informative gene subsets obtained by different feature-selection methods are very minute overlap [36]. However, different models combined with a certain feature-selection method and a suitable classifier can get a close prediction precision [37]. It is difficult to determine which feature-selection method is better. Therefore, evaluation of the robustness of feature-selection methods deserves more attention [32]. In this paper, we analyzed the robustness of *χ* ^2^-IRG-DC by comparing the generalization performance of different models, the efficiency of different feature-selection methods, and the precision of different classifiers.

## 2. Data and Methods

### 2.1. Data

Because nine common binary-class tumor-genomics datasets [28] did not offer independent test sets, we simply selected ten multiclass tumor-genomics datasets with independent test sets (Table 1) for analysis in this study. It should be noted that the method proposed in this paper could also be applied to binary-class datasets.

### 2.2. Weighted Integrated Rank of Genes

Assume the training dataset has *p* markers and *n* samples. The data can be denoted as (*y*_*i*_, *x*_*ij*_) (*i* = 1,…, *n*; *j* = 1,…, *p*). *x*_*ij*_ represents the expression value of the *j*th marker in the *i*th sample;*y*_*i*_ represents the label of *i*th sample, where *y*_*i*_ ∈ *C* = {*C*_1_,…, *C*_*m*_}, the set of possible labels; *m* stands for the total number of labels in the data.

(1)* Chi-Square Values of Single Genes*. For any single gene *G*_*j*_, ${\bar{x}}_{\cdot j}$ denotes the mean expression value of all samples. *Sf*_*k*1_ and *Sf*_*k*2_  (*k* = 1,…, *m*) represent the frequency counts of samples in class *C*_*k*_ when $x_{ij}>{\bar{x}}_{\cdot j}$ and $x_{ij}<{\bar{x}}_{\cdot j}$, respectively. These frequencies can be presented as an  *m* × 2 contingency table, as shown in Table 2. Record the frequency counts of samples in class *C*_*k*_ as *Sf*_*k*3_ When *x*_*ij*_ equals ${\bar{x}}_{\cdot j}$ in class *C*_*k*_, then both *Sf*_*k*1_and *Sf*_*k*2_ should be incremented by 0.5∗*Sf*_*k*3_ separately; thus, the chi-square value *χ*_*j*_^2^ of gene *G*_*j*_ can be calculated according to (1)

(1) $$\begin{array}{c} \chi_{j}^{2}=SN\left(\overset{m}{\underset{k=1}{\sum }}\overset{2}{\underset{q=1}{\sum }}\frac{{Sf}_{kq}^{2}}{{Sn}_{k}{ST}_{q}}-1\right). \end{array}$$

(2)* Chi-Square Values of Pairwise Genes*. For any two genes *G*_*j*_ and *G*_*l*_  (*j* = 1,…, *p*; *l* = 1,…, *p*; *l* ≠ *j*), *Pf*_*k*1_ and *Pf*_*k*2_  (*k* = 1,…, *m*) represent the frequency counts of samples in class *C*_*k*_ when *x*_*ij*_ > *x*_*il*_ and *x*_*ij*_ < *x*_*il*_, respectively. *x*_*ij*_ and *x*_*il*_ are expression values of the *i*th sample in genes *G*_*j*_ and *G*_*l*_, respectively. These frequencies can be presented as an *m* × 2 contingency table (Table 3). Record the frequency counts of samples in class *C*_*k*_ as *Pf*_*k*3_ When *x*_*ij*_ equals *x*_*il*_ in class *C*_*k*_, then both *Pf*_*k*1_ and *Pf*_*k*2_ should be incremented by 0.5∗*Pf*_*k*3_ separately. The Chi-square value *χ*_*j*,*l*_^2^ of pairwise genes (*G*_*j*_, *G*_*l*_) can be calculated according to (2)

(2) $$\begin{array}{c} \chi_{j,l}^{2}=PN\left(\overset{m}{\underset{k=1}{\sum }}\;\overset{2}{\underset{q=1}{\sum }}\frac{{Pf}_{kq}^{2}}{Pn_{k}PT_{q}}-1\right). \end{array}$$

(3)* Rank Genes according to Integrated Weighted Score*. Judging whether a gene is important not only should take main effect of gene into account, but also consider the interaction between it and other genes. Therefore, we integrated the Chi-square value of single gene and the Chi-square values of pairwise genes to define an integrated weighted score of each gene *S*_*j*_ as shown in (3). *S*_*j*_ is the integrated weighted score of gene *G*_*j*_  (*j* = 1,…, *p*), *χ*_*j*_^2^ is the chi-square value of single gene *G*_*j*_, and *χ*_*j*,*l*_^2^ is the chi-square value of pairwise genes *G*_*j*_ and *G*_*l*_  (*l* = 1,…, *p*; *l* ≠ *j*). Genes are ranked by the integrated weighted score *S*_*j*_ to become a descending-range sequence. Consider

(3) $$\begin{array}{c} {\text{S}}_{j}=\chi_{j}^{2}+\overset{p}{\underset{l=1}{\sum }}\left(\frac{\chi_{j}^{2}}{\chi_{j}^{2}+\chi_{l}^{2}}\times \chi_{j,l}^{2}\right) \end{array}$$

make an ordered list Θ of all the genes *G*_*j*_ in accordance with the descending values of the scores *S*_*j*_.

### 2.3. Chi-Square Test-Based Direct Classifier (*χ* ^2^-DC) 

When the training set has *n* samples and *m* labels, with *r*  (*r* ≥ 2) selected genes, there are *r* × (*r* − 1)/2 contingency tables included, each of which has *m* rows and 2 columns (Table 2). If the testing sample belongs to class *C*_*k*_  (*k* = 1,…, *m*), *r* × (*r* − 1)/2 chi-square values of pairwise genes with *n* + 1 samples (i.e., including *n* training samples and a testing sample) can be worked out. The sum of *r* × (*r* − 1)/2 chi-square values was set as *χ*_(*C*_*k*_)_^2^  (*k* = 1,…, *m*). We assign the test sample to the class with the largest chi-square value: class of testing sample = arg max⁡_*k*=1,…,*m*_ *χ*_(*C*_*k*_)_^2^ [31].

### 2.4. Introduce Ranked Genes Sequentially and Remove Redundant Parts to Obtain Informative Genes

Take the top two genes from the ordered list Θ and extract their expression values from the training dataset to form the initial training set. Next, compute the LOOCV accuracy of the initial training data based on *χ* ^2^-DC and denote it as LOOCV_2_. Record *m* chi-square values *χ*_(*C*_1_)_^2^, *χ*_(*C*_2_)_^2^,…, *χ*_(*C*_*m*_)_^2^ of every sample taken as a measured sample. Finally, introduce parameter *h*, as shown in (4)

(4) $$\begin{array}{c} h=\overset{m}{\underset{k=1}{\sum }}\frac{\chi_{\left(C_{t}\right)}^{2}-\chi_{\left(C_{k}\right)}^{2}}{\chi_{\left(C_{t}\right)}^{2}}\;k\neq t, \end{array}$$

where *C*_*t*_ is the true label of the measured sample. The average value of every training sample is denoted as $\bar{h_{2}}$.

Now import the third gene from the ordered list Θ and extract its expression values from the training dataset to update the initial training set. Following the steps documented above, obtain LOOCV_3_ and $\bar{h_{3}}$ of the updated training set. If LOOCV_3_ > LOOCV_2_, or LOOCV_3_ = LOOCV_2_ and $\bar{h_{3}}>\bar{h_{2}}$, the third gene is selected as an informative gene; Otherwise, it is deemed as a redundant gene.

Similarly, informative gene subsets will be obtained by sequentially introducing the top 2% genes from the ordered list Θ.

### 2.5. Independent Prediction

With the informative gene subsets, independent prediction based on *χ* ^2^-DC was conducted individually on the testing sample to obtain the test accuracy.

### 2.6. Models Used for Comparison

In this paper, a model is considered as a combination of a specific feature-selection method and a specific classifier. Some feature-selection methods are also classifiers (HC-TSP, HC-*k*-TSP, TSG, DT, PAM, etc.). We selected mRMR-SVM, SVM-RFE-SVM, HC-*k*-TSP and TSG as comparative models for *χ* ^2^-IRG-DC; NB, KNN, and SVM as the comparative classifiers of *χ* ^2^-DC; mRMR, SVM-RFE, HC-*k*-TSP and TSG as the comparative feature-selection approaches of *χ* ^2^-IRG-DC.

mRMR conducts minimum redundancy maximum relevance feature selection. Mutual information difference (MID) and mutual information quotient (MIQ) are two versions of mRMR. MIQ was better than MID in general [9], so the evaluation criterion in this paper is mRMR-MIQ. SVM-RFE is a simple and efficient algorithm which conducts gene selection in a backward elimination procedure. The mRMR and SVM-RFE have been widely applied in analyzing high-dimensional biological data. They only provide a list of ranked genes; a classification algorithm needs to be used to choose the set of variables that minimize cross validation error. In this paper, SVM was selected as the classification algorithm, and our SVM implementation is based on LIBSVM which supports 1-versus-1 multiclass classification. For SVM-RFE-SVM and mRMR-SVM models, informative genes were selected by the following methods: (i) rank the genes separately by mRMR or SVM-RFE; (ii) select the top genes from 1 to *s*, which is equal to approximately 2% of the total gene number, and conduct 10-fold cross-validation (CV10) for the training sets based on SVM. Accuracy was denoted as CV10_*w*_  (*w* = 1,…, *s*); (iii) with the highest CV10 accuracy, the genes were selected as informative genes.

## 3. Results and Discussion

### 3.1. Comparison of Independent Test Accuracy and the Number of Informative Genes Used in Different Models

In order to evaluate the performance of model in this study, we used the eight different models to perform independent test on ten multiclass datasets. The test accuracy and informative gene number are presented in Table 4. In this case, the classification accuracy of each dataset is the ratio of the number of the correctly classified samples to the total number of samples in that dataset. The best model based on average accuracy of the ten multiclass datasets used in this study is *χ* ^2^-IRG-DC (90.81%), followed by TSG (89.2%), PAM (88.5%), SVM-RFE-SVM (86.72%) and HC-*k*-TSP (85.12%). We do not consider these differences in accuracy as noteworthy and conclude that all five methods perform similarly. However, in terms of efficiency, decision rule and the number of informative genes, one can argue that the *χ* ^2^-IRG-DC method is superior. Recall that the *χ* ^2^-IRG-DC, TSG and PAM have easy interpretation and can directly handle multiclass case, but HC-*k*-TSP and SVM-RFE-SVM need a tedious process to covert multiclass case into binclass case. For the ten multiclass datasets, *χ* ^2^-IRG-DC selected 37.2 (range, 20–64 in ten datasets) informative genes on average. It clearly uses less number of genes than PAM (1638.8) and TSG (51). Moreover, the algorithm complexities of *χ* ^2^-IRG-DC is far less than TSG. *χ* ^2^-IRG-DC ranked all genes according to integrated weighted score firstly and sequentially introduced the ranked genes based on LOOCV accuracy of training data. In fact, *χ* ^2^-IRG-DC is a hybrid filter-wrapper models that take advantage of the simplicity of the filter approach for initial gene screening and then make use of the wrapper approach to optimize classification accuracy in final gene selection [38].

### 3.2. Robustness Analysis—Evaluating Generalization Performance of Different Models

**Figure 1:**
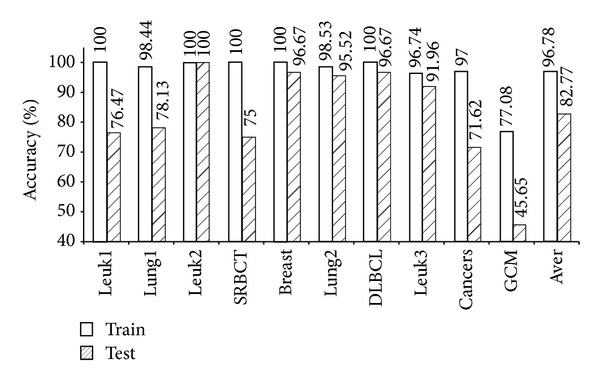
Accuracy of mRMR-SVM for training and test data.

**Figure 2:**
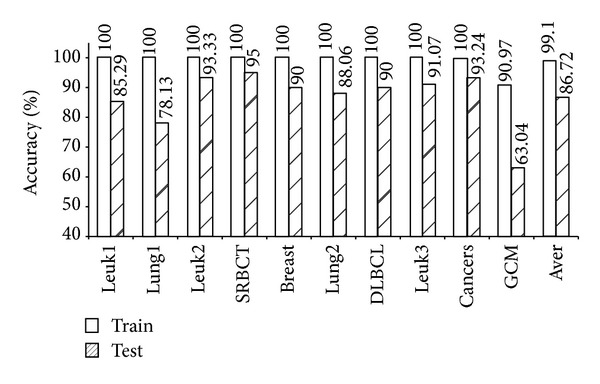
Accuracy of SVM-RFE-SVM for training and test data.

**Figure 3:**
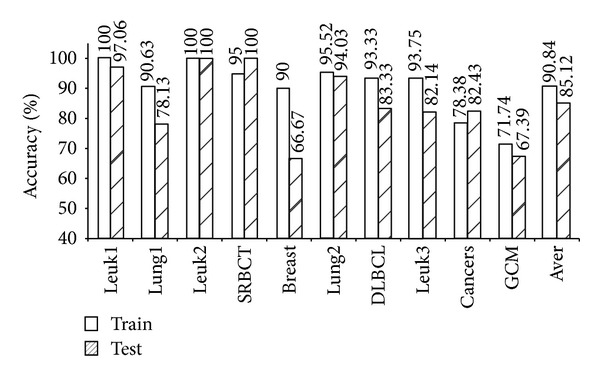
Accuracy of HC-*k*-TSP for training and test data.

**Figure 4:**
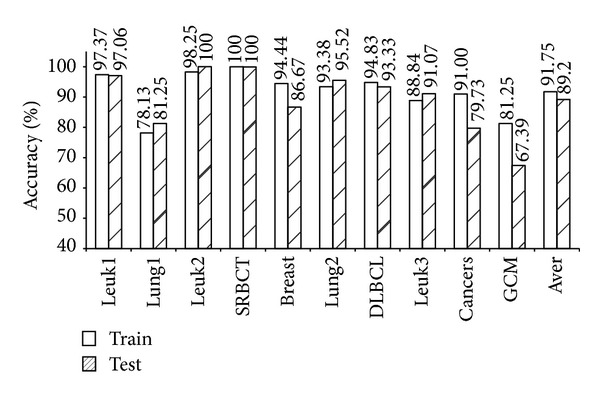
Accuracy of TSG for training and test data.

**Figure 5:**
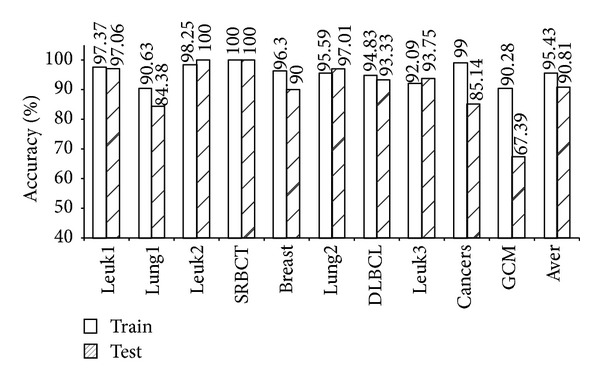
Accuracy of *χ* ^2^-IRG-DC for training and test data.

As shown in Table 4, the five models (mRMR-SVM, SVM-RFE-SVM, HC-*k*-TSP, TSG, and *χ* ^2^-IRG-DC) exhibited high independent test accuracy and similar informative gene numbers. We further compared the LOOCV accuracy for the training data and the independent test accuracy for the test data from these four models. The results are shown in Figures 1, 2, 3, 4, and 5. Obviously, over-fitting occurred in all five models. Among them, *χ* ^2^-IRG-DC had higher generalization performance. The test accuracy of mRMR-SVM and SVM-RFE-SVM was no greater than their training accuracy for all ten datasets. However, the test accuracy of *χ* ^2^-IRG-DC was superior to the training accuracy for the Leuk2, Lung2, and Leuk3 datasets, and the test accuracy of TSG was superior to the training accuracy for the Lung1, Lung2, Leuk2, and Leuk3 datasets. For another direct classifier, HC-*k*-TSP, the test accuracy was also higher than the training accuracy for the SRBCT and cancers datasets. These results indicated that the special direct classification algorithm of *χ* ^2^-IRG-DC, TSG and HC-*k*-TSP can effectively control over-fitting, and exhibiting a better generalization performance.

### 3.3. Robustness Analysis—Evaluating Different Feature-Selection Methods

As shown in Table 5, with the informative genes selected by the five feature-selection methods, the classification performances of NB and KNN were significantly improved. However, the performance of SVM was improved only with the genes selected by our method, *χ* ^2^-IRG-DC. This observation indicated, on the one hand, that SVM is not sensitive to feature dimensions [39], and on the other hand, that *χ* ^2^-IRG-DC was more robust than the other four feature-selection methods.

With the genes selected by *χ* ^2^-IRG-DC, four classifiers (NB, KNN, SVM, and *χ* ^2^-DC) performed very well, with average accuracies of 84.23%, 85.54%, 89.54%, and 90.81%, respectively, across ten datasets; the overall average accuracy was 87.53%. Similarly, we calculated the overall average accuracy of other feature-selection methods: 87.53% (*χ* ^2^-IRG-DC) > 85.99% (HC-*k*-TSP) > 84.45% (TSG) > 81.93% (SVM-RFE) > 80.16% (mRMR), once again confirming the robustness and effectiveness of *χ* ^2^-IRG-DC.

### 3.4. Robustness Analysis—Comparison of Classifiers

The overall average accuracies of the four classifiers with informative genes selected by five feature-selection methods across ten datasets are highlighted in bold in Table 5. The order is as follows: 85.86% (SVM) > 85.51% (*χ* ^2^-DC) > 83.42% (NB) > 81.24% (KNN). This result revealed that SVM is an excellent classifier; at the same time, the *χ* ^2^-DC classifier also performed well.

## 4. Conclusion

Informative gene subsets selected by different feature-selection methods often differ greatly. As we can see, genes number selected by the three different models (mRMRSVM, SVM-RFE-SVM) in are listed in Table S1. The numbers of overlapped gene selected by different models are listed in Table S2. Results showed that there are few overlaps of genes selected by the three models (see supplementary Tables S1 and S2 in supplementary materials available online at http://dx.doi.org/10.1155/2014/589290). However, different models combined with a certain feature-selection method and a suitable classifier can get a close prediction precision. Evaluations of robustness of feature-selection methods and classifiers should include the following aspects: (i) models should have good generalization performance, that is, a model should not only have high accuracy in training sets, but should also have high and stable test accuracy across many datasets (average accuracy ± standard deviation); (ii) with informative genes selected by an excellent feature-selection method, should improve varies classifiers performance; (iii) similarly, a good classifier should perform well with different informative genes selected by different excellent feature-selection approaches.

The results of this study illustrate that pairwise interaction is the fundamental type of interaction. Theoretically, the complexity of the algorithm could be controlled within *O*(*n* ^2^) with pairwise interactions. When three or more genes connect to each other, the complex combination of three or more genes could be represented by the pairwise interactions. Based on this assumption, this paper proposes a novel algorithm, *χ* ^2^-IRG-DC, used for informative gene selection and classification based on chi-square tests of pairwise gene interactions. The proposed method was applied to ten multiclass gene-expression datasets; the independent test accuracy and generalization performance were obviously better than those of mainstream comparative algorithms. The informative genes selected by *χ* ^2^-IRG-DC were able to significantly improve the independent test accuracy of other classifiers. The average extent of test accuracy raised by *χ* ^2^-IRG-DC is superior to those of comparable feature-selection algorithms. Meanwhile, informative genes selected by other feature-selection methods also performed well on *χ* ^2^-DC.

Currently, integrated analysis of multisource heterogeneous data is a key challenge in cancer classification and informative gene selection. This includes the integration of repeated measurements from different assays for the same disease on the same platform [40], as well as the integration of gene chips, protein mass spectrometry, DNA methylation, and GWAS-SNP data collected on different platforms for the study of the same disease [41], and so forth. In future, we will apply *χ* ^2^-IRG-DC to the integrated analysis of multi-source heterogeneous data. Combining this method with the GO database, biological pathways, disease databases, and relevant literature, we will conduct a further assessment of the relevance of the biological functions of selected informative genes to the mechanisms of disease [42].

## Supplementary Material

Table S1: The number of genes selected by the different models.
Table S2: Overlaps of genes selected by different models.

## Figures and Tables

**Table 1:** Multiclass gene-expression datasets.

Dataset	Platform	No. of classes	No. of genes	No. of samples in training	No. of samples in test	Source
Leuk1	Affy	3	7,129	38	34	[6]
Lung1	Affy	3	7,129	64	32	[43]
Leuk2	Affy	3	12,582	57	15	[44]
SRBCT	cDNA	4	2,308	63	20	[45]
Breast	Affy	5	9,216	54	30	[46]
Lung2	Affy	5	12,600	136	67	[47]
DLBCL	cDNA	6	4,026	58	30	[48]
Leukemia3	Affy	7	12,558	215	112	[49]
Cancers	Affy	11	12,533	100	74	[50]
GCM	Affy	14	16,063	144	46	[51]

**Table 2:** Frequency counts of samples in each class for single genes.

Class	$x_{ij}>{\bar{x}}_{∙j}$	$x_{ij}<{\bar{x}}_{∙j}$	Total
*C*_1_	*Sf*_11_	*Sf*_12_	*Sn*_1_ = *Sf*_11_ + *Sf*_12_
⋮	⋮	⋮	⋮
*C*_*m*_	*Sf*_*m*1_	*Sf*_*m*2_	*Sn*_*m*_ = *Sf*_*m*1_ + *Sf*_*m*2_

Total	$ST_{1}=\overset{m}{\underset{k=1}{\sum }}{Sf}_{k1}$	$ST_{2}=\overset{m}{\underset{k=1}{\sum }}{Sf}_{k2}$	$SN=\overset{m}{\underset{k=1}{\sum }}{Sn}_{k}$

**Table 3:** Frequency counts of samples in each class for pairwise genes.

Class	x_*ij*_ > x_*il*_	x_*ij*_ < x_*il*_	Total
*C*_1_	*Pf*_11_	*Pf*_12_	*Pn*_1_ = *Pf*_11_ + *Pf*_12_
⋮	⋮	⋮	⋮
*C*_*m*_	*Pf*_*m*1_	*Pf*_*m*2_	*Pn*_*m*_ = *Pf*_*m*1_ + *Pf*_*m*2_

Total	$PT_{1}=\overset{m}{\underset{k=1}{\sum }}Pf_{k1}$	$PT_{2}=\overset{m}{\underset{k=1}{\sum }}Pf_{k2}$	$PN=\overset{m}{\underset{k=1}{\sum }}Pn_{k}$

**Table 4:** Independent test accuracy and informative gene number used indifferent models (in parentheses) for multiclass gene-expression datasets.

Model	Leuk1	Lung1	Leuk2	SRBCT	Breast	Lung2	DLBCL	Leuk3	Cancers	GCM	Aver ± std
HC-TSP∗	**97.06**	71.88	80	95	66.67	83.58	83.33	77.68	74.32	52.17	78.17 ± 13.17
	(4)	(4)	(4)	(6)	(8)	(8)	(10)	(12)	(20)	(26)	(10.2)

HC-K-TSP∗	**97.06**	78.13	**100**	**100**	66.67	94.03	83.33	82.14	82.43	**67.39**	85.12 ± 12.42
	(36)	(20)	(24)	(30)	(24)	(28)	(46)	(64)	(128)	(134)	(53.4)

DT∗	85.29	78.13	80	75	73.33	88.06	86.67	75.89	68.92	52.17	76.35 ± 10.49
	(2)	(4)	(2)	(3)	(4)	(5)	(5)	(16)	(10)	(18)	(6.9)

PAM∗	**97.06**	78.13	93.33	95	**93.33**	**100**	90	**93.75**	87.84	56.52	88.5 ± 12.71
	(44)	(13)	(62)	(285)	(4,822)	(614)	(3,949)	(3,338)	(2,008)	(1,253)	(1,638.8)

mRMR-SVM	76.47	78.13	100.00	75.00	96.67	95.52	96.67	91.96	71.62	45.65	82.77 ± 16.85
	(7)	(13)	(19)	(9)	(97)	(120)	(16)	(119)	(89)	(57)	(54.6)

SVM-RFE-SVM	85.29	78.13	93.33	95.00	90.00	88.06	90.00	91.07	93.24	63.04	86.72 ± 9.62
	(5)	(9)	(8)	(3)	(7)	(9)	(13)	(35)	(29)	(199)	(31.7)

TSG	97.06	81.25	100	100	86.67	95.52	93.33	91.07	79.73	67.39	89.20 ± 10.5
	(6)	(20)	(44)	(13)	(63)	(60)	(16)	(95)	(81)	(112)	(51)

*χ* ^2^-IRG-DC	**97.06**	84.38	**100**	**100**	90	97.01	93.33	**93.75**	85.14	**67.39**	**90.81** ± 9.91
	(29)	(23)	(20)	(23)	(31)	(52)	(37)	(46)	(47)	(64)	(37.2)

*Results reported in [28].

**Table 5:** Test accuracy of different classifiers with informative genes selected by different feature-selection methods.

Classifier	Feature-selection method	Leuk1	Lung1	Leuk2	SRBCT	Breast	Lung2	DLBCL	Leuk3	Cancers	GCM	Aver-*F*
NB	ALL∗	85.29	81.25	100.00	60.00	66.67	88.06	86.67	32.14	79.73	52.17	73.20
	*χ* ^2^-IRG-DC	97.06	81.25	100.00	85.00	86.67	92.54	96.67	59.82	82.43	60.87	84.23
	mRMR	79.41	68.75	100.00	90.00	93.33	97.01	96.67	74.11	70.27	45.65	81.52
	SVM-RFE	67.65	81.25	80.00	95.00	80.00	89.55	90.00	95.00	77.03	63.04	81.85
	HC-K-TSP	91.18	81.25	100.00	80.00	80.00	95.52	86.67	100.00	77.03	65.22	85.69
	TSG	91.18	84.38	93.33	100	86.67	94.03	100	51.79	71.62	65.22	83.82
	Aver-*C* ^†^	85.30	79.38	94.67	90.00	85.33	93.73	94	76.14	75.68	60.00	**83.42**

KNN	ALL∗	67.65	75.00	86.67	70.00^‡^	63.33	88.06	93.33	75.89	64.86	34.78	71.96
	*χ* ^2^-IRG-DC	97.06	71.88	86.67	100.00	86.67	85.07	96.67	87.50	85.14	58.70	85.54
	mRMR	70.59	68.75	80.00	80.00	96.67	86.57	100.00	91.07	54.05	36.96	76.47
	SVM-RFE	76.47	68.75	86.67	100.00	90.00	86.57	90.00	91.96	58.11	45.65	79.42
	HC-K-TSP	88.24	87.50	86.67	85.00	83.33	94.03	93.33	88.39	64.86	52.17	82.35
	TSG	91.18	75	93.33	100	80	88.06	96.67	86.6	74.32	39.13	82.43
	Aver-*C* ^†^	84.71	74.38	86.67	93.00	87.33	88.06	95.33	89.10	67.30	46.52	**81.24**

SVM	ALL∗	79.41	87.50	100.00	100.00	83.33	97.01	100.00	84.82	83.78	65.22	88.11
	*χ* ^2^-IRG-DC	97.06	87.50	93.33	100.00	93.33	92.54	96.67	86.61	91.89	56.52	89.54
	mRMR	76.47	78.13	100.00	75.00	96.67	95.52	96.67	91.96	71.62	45.65	82.77
	SVM-RFE	85.29	78.13	93.33	95.00	90.00	88.06	90.00	91.07	93.24	63.04	86.72
	HC-K-TSP	85.29	84.38	100.00	90.00	86.67	98.51	96.67	94.64	82.43	60.87	87.95
	TSG	91.18	81.25	93.33	80	80	94.03	100	80.36	68.92	54.35	82.34
	Aver-*C* ^†^	87.06	81.88	96.00	88.00	89.33	93.73	96.00	88.93	81.62	56.09	**85.86**

*χ* ^2^-DC	*χ* ^2^-IRG-DC	97.06	84.38	100.00	100.00	90.00	97.01	93.33	93.75	85.14	67.39	90.81
	mRMR	82.35	65.63	100.00	90.00	90.00	95.52	70.00	96.43	60.81	47.83	79.86
	SVM-RFE	79.41	56.25	66.67	85.00	76.67	92.54	80.00	96.43	94.59	69.57	79.71
	HC-K-TSP	97.06	84.38	100.00	95.00	76.67	97.01	93.33	88.39	78.38	69.57	87.98
	TSG	97.06	81.25	100	100	86.67	95.52	93.33	91.07	79.73	67.39	89.20
	Aver-*C* ^†^	90.59	74.38	93.33	94.00	84.00	95.52	86.00	93.21	79.73	64.35	**85.51**

*Results reported in [28]; ^‡^30 in original paper, whereas the actual number was 70 after validation; ^†^Aver-*C* was the average accuracy of a classifier with informative genes selected by four feature-selection methods.
