# Supplementary material for: Informative Gene Selection and Direct Classification of Tumor Based on Chi-Square Test of Pairwise Gene Interactions
Source: Biomed Res Int. 2014 Jul 23;2014:589290. doi: 10.1155/2014/589290 (PMC4130026; doi:10.1155/2014/589290)
Supplement: Supplementary file 1 — Table S1: The number of genes selected by the different models. Table S2: Overlaps of genes selected by different models. [file 589290.f1.zip › mat.589290.v2/additional file 2/chi-IRG-DC/readme.doc]

```
*********************************************************
*							                                       *
*  README 						                                   *
*  							                                       *
*  Hongyan Zhang <hongyan_zhang6@aliyun.com>	           *
*							                                       *
*  Version: July 2013 	                                       *
*********************************************************

CONTENTS:
The folder contains 7 programs:
(The '.m' is matlab script or matlab function file)
1) get_chi_square_single.m ( get the chi-square values of single genes)
2) get_chi_square.m (get the chi-square values of pairwise genes )
3) get_chi_weight_rank.m (rank genes according to integrated weighted score )
4) get_top_gain_tsn.m (Introduce ranked genes sequentially and remove redundant parts to obtain informative genes)
```

5) tsn_loocv_rate.m ( compute the LOOCV accuracy based on $\chi^{2}$-DC )

6) tsn_predict.m (compute independent prediction accuracy based on $\chi^{2}$-DC )

7) go_Chi_IRG_DC.m	(an example script for $\chi^{2}$-IRG-DC )

```
The folder contains 2 datasets:
leuk1_train.mat   (a training data as an example)
leuk1_test.mat    (a test data as an example)
```
